# Impact of growth media and pressure on the diversity and antimicrobial activity of isolates from two species of hexactinellid sponge

**DOI:** 10.1099/mic.0.001123

**Published:** 2021-12-13

**Authors:** Matthew J. Koch, Poppy J. Hesketh-Best, Gary Smerdon, Philip J. Warburton, Kerry Howell, Mathew Upton

**Affiliations:** ^1^​ School of Biomedical Sciences, University of Plymouth, Plymouth PL4 8AA, UK; ^2^​ School of Biological and Marine Sciences, University of Plymouth, Plymouth PL4 8AA, UK; ^3^​ Diving Diseases Research Centre Healthcare, Plymouth Science Park, Research Way, Plymouth PL6 8BU, UK

**Keywords:** sponge, natural product discovery, hexactinellid, antimicrobial, culture

## Abstract

Access to deep-sea sponges brings with it the potential to discover novel antimicrobial candidates, as well as novel cold- and pressure-adapted bacteria with further potential clinical or industrial applications. In this study, we implemented a combination of different growth media, increased pressure and high-throughput techniques to optimize recovery of isolates from two deep-sea hexactinellid sponges, *Pheronema carpenteri* and *Hertwigia* sp., in the first culture-based microbial analysis of these two sponges. Using 16S rRNA gene sequencing for isolate identification, we found a similar number of cultivable taxa from each sponge species*,* as well as improved recovery of morphotypes from *P. carpenteri* at 22–25 °C compared to other temperatures, which allows a greater potential for screening for novel antimicrobial compounds. Bacteria recovered under conditions of increased pressure were from the phyla *Proteobacteria *, *Actinobacteria* and *Firmicutes *, except at 4 %O_2_/5 bar, when the phylum *Firmicutes* was not observed. Cultured isolates from both sponge species displayed antimicrobial activity against *Micrococcus luteus, Staphylococcus aureus* and *Escherichia coli *.

## Introduction

The worldwide threat to public health posed by drug-resistant bacteria has become increasingly apparent over the last decade, with a seminal UK report predicting that deaths attributable to drug-resistant bacteria will rise from 700 000 per year to 10 million by 2050 [1]. Additionally, issues with the supply chain of new antibiotics has become a major contributor to the inability to treat microbial infections [2]. Prior to the discovery of teixobactin, identified through the employment of novel culture techniques [3], no novel class of antibiotics had been discovered in several decades. Most current antibiotics have been derived from the natural environment [2], and while it will continue to be an important source for future compounds, there is a need to innovate by investigating underexplored areas and improving culture methodology.

Sponges (Porifera) are sessile metazoan organisms thought to have emerged around 600 million years ago [4, 5]. As filter-feeding organisms, certain species have been predicted to filter up to 50 000 litres of seawater per litre of sponge per day [6], bringing them into contact with large quantities of marine debris, nutrients and planktonic bacteria. Representing the most widely sampled marine phyla in the hunt for novel bioactives over the last 45 years, the Porifera comprise the most prolific source of such agents from the marine environment [7, 8]. Both culture-dependent and culture-independent studies have been used to reveal the interspecific and intraspecific differences between the microbiota of different sponge species, as well as their antimicrobial potential [9–11].

The cultivation of sponge-associated microbes has traditionally been limited by poor access to samples [12] or difficulty in providing suitable culture parameters [13, 14]. Efforts to improve the cultivation have included an analysis of different methodologies, such as agar-based recovery [10], the use of diffusion chambers [15], liquid culture and floating-filter cultivation [16]. Agar-based methods have shown greater success in cultivating an increased bacterial diversity from *Haliclona* species, compared to liquid and floating-filter methods [16]. *In situ* implantation of diffusion growth chambers (DGCs) within the living tissue of *Rhabdastrella globostellata* sponge have also resulted in the improved recovery of bacteria belonging to the *Actinobacteria *, *Alphaproteobacteria* and *Gammaproteobacteria* [15], many of which were deemed to be novel. This represents a promising method for the cultivation of bacteria from larger, shallow-water sponges, but the *in situ* nature of the methodology would make implementation on deep-sea sponges more problematic.

For every depth increase of 10.06 m, pressure increases one atmosphere. Therefore, the retrieval of sponge samples from deeper waters brings with it the potential to isolate bacteria adapted to life at both lower temperatures and higher pressures. Bacteria adapted to survive in such conditions can be separated into several categories. Piezotolerant bacteria are those capable of surviving at increased atmospheric pressures, but at which their optimal growth does not occur. In contrast, piezophilic bacteria grow more favourably at higher pressure, while hyperpiezophilic are those that only grow at increased atmospheric pressure [17]. Whilst research has revealed the extent to which piezotolerant/piezophilic bacteria participate in ecological cycles such as nutrient cycling and degradation [18], it has been remarked upon that the effect of host–microbe interactions on the ability of bacteria to thrive under increased atmospheric pressures is poorly understood [19]. To the best of our knowledge, the use of increased atmospheric pressure to improve the cultivability of sponge bacteria has not yet been explored.

Hexactinellid, or ‘glass’, sponges represent an extremely under-explored class of Porifera, in relation to their microbiota and the bioactive potential of associated microbes [20, 21]. Occurring almost exclusively below 200 m, hexactinellid sponges are characterized by a ‘fused’, or basket-like skeleton, consisting of siliceous spicules. Most culture-dependent studies to date have focused on sponges obtained from shallow waters, predominately those of the Demosponge population. Mangano *et al*. [20], however, first reported the recovery of bacterial isolates from a deep-sea hexactinellid sponge (*Anoxycalyx joubini*), in a study that included both demosponges and the hexactinellids. Similarly, Xin *et al*. [21] reported the cultivation of bacterial isolates from two species of Hexactinellid sponges, *Rossella nuda* and *Rossella racovitzae*, including the cultivation of a potentially novel group of previously uncultured isolates. This suggests a potentially distinct hexactinellid-specific microbiota and is further supported by recent 16S rRNA amplicon sequencing and metagenomic surveys [22–24]. The evidence that bacterial genomes associated with *Vazella pourtalesii* display genome reduction is potentially indicative of specialized, hexactinellid-specific host–microbe interactions [23]. These reports highlight a potentially diverse but uncharacterized microbiome specific to individual sponge species, which opens up an opportunity to investigate this unique flora for novel antimicrobial compounds.

The current lack of information regarding the cultivable sponge-associated inhabitants of Hexactinellid sponges and their antimicrobial potential represents a knowledge gap in the current literature. Therefore, the aim of this study was to utilize novel media and pressure culture methods to improve recovery of hexactinellid-associated bacteria in the search for novel antimicrobial producers.

## Methods

### Sample collection

**Fig. 1.**
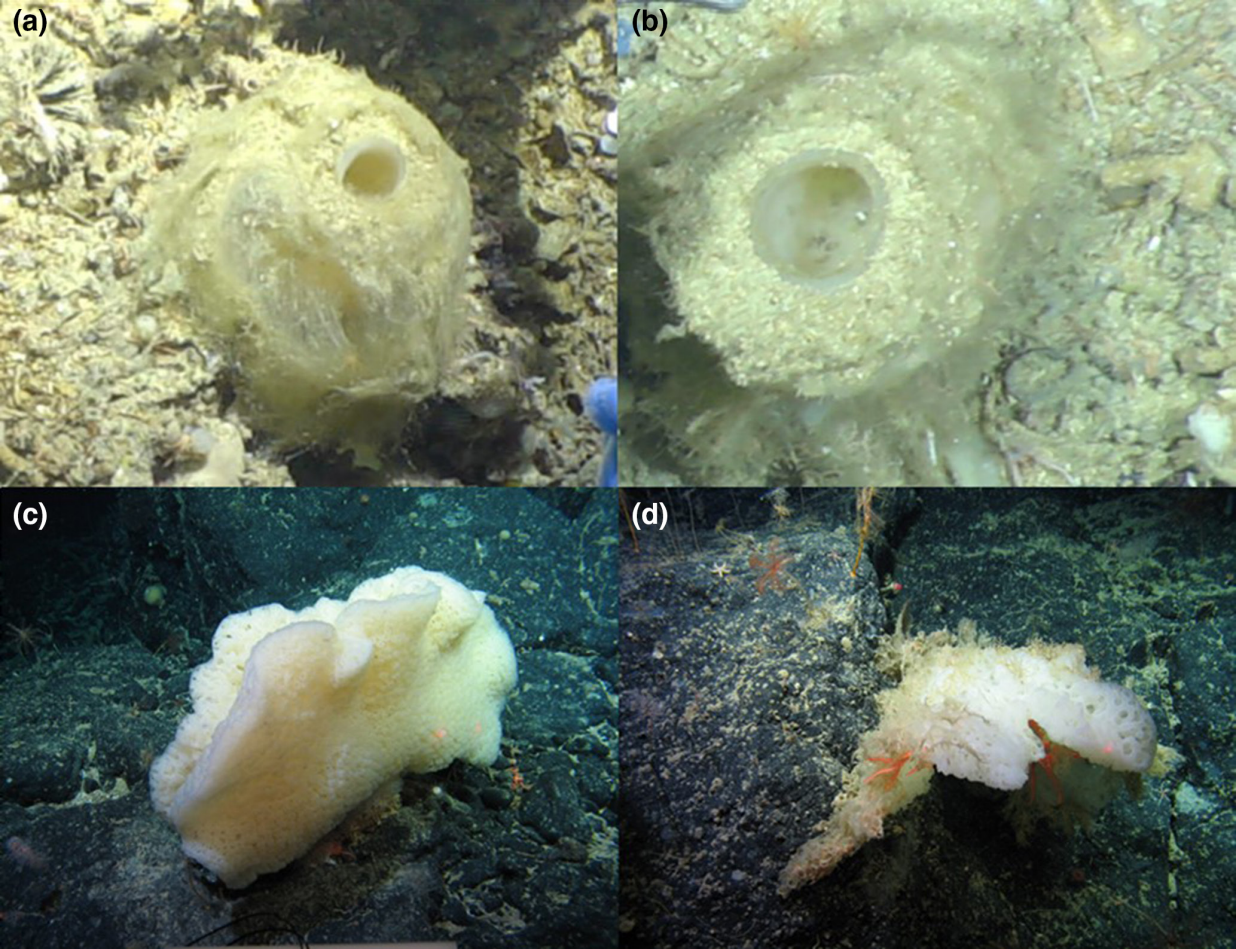
Photos of sponges taken *in situ* before sample collection. (a, b) *Pheronema carpenteri* sponges (JC136). (c, d) *Hertwigia* sp. sponges (GRNL17).

Sponge samples were collected from the North East Atlantic deep-sea, as part of two different research programmes: the NERC-funded DeepLinks Project in 2016 (RRS *James Cook* - JC136), and the Sensitive Ecosystem Assessment and ROV Exploration of Reef (SEAROVER) project in 2017 (Irish Light Vessel *Granuaile* - GRNL2017, RH17001), and again in 2019 (RV *Celtic Explorer* CE19015). Sample collection sites are displayed in Fig. S4 (available in the online version of this article) with sample co-ordinates and metadata displayed in Table S3. Both cruises conducted sampling of a wide range of deep-sea organisms. Sponges were collected by remotely operated vehicles (ROVs) and photographed *in situ* before removal (see Fig. 1).

On surfacing, sponges were transferred from the ROV into buckets containing *in situ* seawater and taken into the laboratory for processing. Sponge samples were photographed and a small tissue sample was taken for genetic analysis. The remaining sponge sample was then placed in a plastic zip-lock bag and frozen at −20 °C for the remainder of the cruise. Upon return to land, frozen samples were transported in dry ice and maintained at −80 °C.

### Sponge identification

Sponges were identified from the analysis of internal and external morphological features (i.e. body shape, type, size and arrangement of spicules) following the Systema Porifera classification system [25]. For spicule analysis, under sterile conditions using ethanol-washed and flame-sterilized scalpels, cuttings of approximately 1 cm^3^ were taken from the three regions on the sponge body, mesohyl, atria and the prostalia. Tissues were placed inside Eppendorf tubes (2 ml), covered with 65% nitric acid and left for 2 h for spongin tissue to dissolve. The tubes were gently centrifuged at 600 ***g*** for 2 min. The supernatant was carefully discarded and the pellet containing spicules was re-suspended in water three times to wash all remaining acid. Spicules were then washed in >95% ethanol twice before being left at room temperature for the ethanol to evaporate. Dry spicules were inspected under a compound light microscope and identified [25].

### Sample processing

For isolation of bacteria, sponge samples were allowed to come to room temperature naturally. Sections of the sponge mesohyl were cut from the sponge using a sterile scalpel. Individual tissue segments (~10 g) were then homogenized using a sterile mortar and pestle and transferred to a sterile 50 ml falcon tube (Fisher Scientific). Large and un-degradable (spicular) debris was left to settle for 5 min and the remaining suspended homogenate was transferred to a new sterile 50 ml falcon tube. The homogenate was then centrifuged (4696 ***g**,* 20 min) to obtain a pellet. The pellet was then re-suspended in 2 ml sterile PBS and 100 µl was spread onto individual agar plates. To minimize the effects of repetitive freezing on the original sponge sample, as well as to aid replicability, all sponge segments were processed at the same time. Unused bacterial cell suspensions and sponge tissue segments were stored at −20 °C in natural seawater (NSW) + 50% glycerol for later use.

### Agar-based comparison of bacterial richness and abundance

A range of solid-growth media was used for bacterial recovery. Abbreviations in the text and figures are as follows. MA: marine agar; MC: marine agar+carnitine hydrochloride (0.2 g l^−1^); LB: LB agar; LC: LB agar+carnitine hydrochloride (0.2 g l^−1^); RC: R2A agar+carnitine hydrochloride (0.2 g l^−1^); OM: oatmeal agar; SYP-SW: starch-yeast-peptone-seawater agar [26]; ABC: PS medium [27]. A full list of media used is contained within the Supplementary Material. A bacterial cell suspension was spread evenly across agar plates. Where agar contained carnitine hydrochloride, 0.2 g l^−1^ carnitine hydrochloride (Sigma-Aldrich) was added before autoclaving. Sponge spicule extract (SSE) was prepared as follows: 10 g sponge host tissue was homogenized with a sterilized mortar and pestle. Homogenate was extracted overnight in 50 ml distilled H_2_O (dH_2_O) and filtered-sterilized using a 0.22 µm filter. The cake remaining on filters was suspended in 50 ml dH_2_O, centrifuged at 138 ***g*** for 10 min and the pellet re-suspended in 20 ml dH_2_O. SSE (40 ml l^−1^) was added to 1.5% agar, 33.3 g l^−1^ Instant Ocean and 9.05 g l^−1^ R2A before autoclaving.

For each condition tested, three technical replicates were performed for each biological sponge replicate. For assessing the impact of different media on cultivation, all plates were incubated in the dark at 20 °C for a total of 40 days, when all growth measurements were taken.

For assessing the effect of temperature on cultivation, four temperatures were initially assessed; 4 °C, 15 °C, ambient (22–25 °C) and 28 °C on low nutrient ½ R2A agar. Plates incubated at room temperature or above were incubated for 40 days and inspected bi-weekly. For lower incubation temperatures, the incubation period was 90 days, and cultures were monitored weekly.

For pressurized culture, agar plates spread with bacterial cell suspensions were placed into stainless steel containers (650×300 mm) (Southwestern Engineering). Gas mixtures were prepared at either 4 or 21% O_2_ prior to being pumped into the chambers. Chambers were filled with gas mixtures until the desired pressure had been reached.

### Dilution to extinction – cell counting and dilution

Bacterial cell suspensions from *P. carpenteri* (JC126_125/134) and *Hertwigia* sp. (GRNL_081/082) were prepared according to the steps outlined above. The approximate bacterial concentration was determined with the use of a haemocytometer. Cell suspensions were then diluted to approximately three to four cells per millilitre, and 333 µl was added to each well of a 96-well plate, to give an approximate total of ~1 cell per well. A total of 288 wells were inoculated. Plates were incubated at 20 °C for a period of 40 days.

### Antimicrobial screening using simultaneous antagonism

Cell suspensions of the organism being screened against (the indicator) were prepared to specific OD_600_ readings for each species. Indicators used for preliminary screening were *Micrococcus luteus* (OD_600_ 0.5), *Staphylococcus aureus* NCTC12493 (OD_600_ 0.5) and *Escherichia coli* NCTC10418 (OD_600_ 0.4). Isolates obtained from bacterial culture were screened for antimicrobial activity using the simultaneous antagonism method [28].

### DNA extraction and amplification

Genomic DNA was extracted from isolates using the DNeasy Powersoil Kit (Qiagen) according to the manufacturer’s conditions. DNA was quantified using a Qubit Fluorometer (Thermofisher Scientific). PCRs (50µl) were set up, consisting of 25 µl 2× DreamTaq Green PCR Master Mix (Fisher Scientific), 2.5 µl 27F 16S Primer (5′–3′ AGAGTTTGATCATGGCTCA), 2.5µl 1492R 16S Primer (5′–3′ TACGGTTACCTTGTTACGACTT) (Eurofins Genomics Standard Primers) [29–32], 5µl DNA Template (50–100 ng) and 15µl nuclease-free water (Merck). PCR conditions for amplification of the 16S rRNA gene consisted of an initial denaturation step of 5 min at 94°C, followed by 30 cycles of 30 s at 94 °C, 60 s at 52 °C, 90 s at 72 °C and a final extension step of 10 min at 72 °C.

### Bacterial sequence classification and tree building

Amplicons of the 16S rRNA gene were sequenced using Sanger Sequencing (LGC Genomics). The forward and reverse strands of the 16S rDNA gene were sequenced separately and trimmed to remove regions that had more than a 5% chance of per-base error. Forward and reverse sequences were then aligned to each other in order to provide a consensus sequence. Sequences were compared against the NCBI Nucleotide collection (nr/nt) database using the blast function in Geneious Prime v2020.2.2 with standard parameters. A phylogenetic, neighbour-joining tree was reconstructed using the Tamura–Nei distance model [33]. Tree-building was performed in Geneious Prime using the Geneious Tree Builder tool with standard parameters. The tree was exported as Newick-format and uploaded to iToL (https://itol.embl.de/) for visualization.

## Results

### Low-nutrient media recover highest bacterial abundance and diversity for *P. carpenteri* and *Hertwigia* sp.

**Fig. 2.**
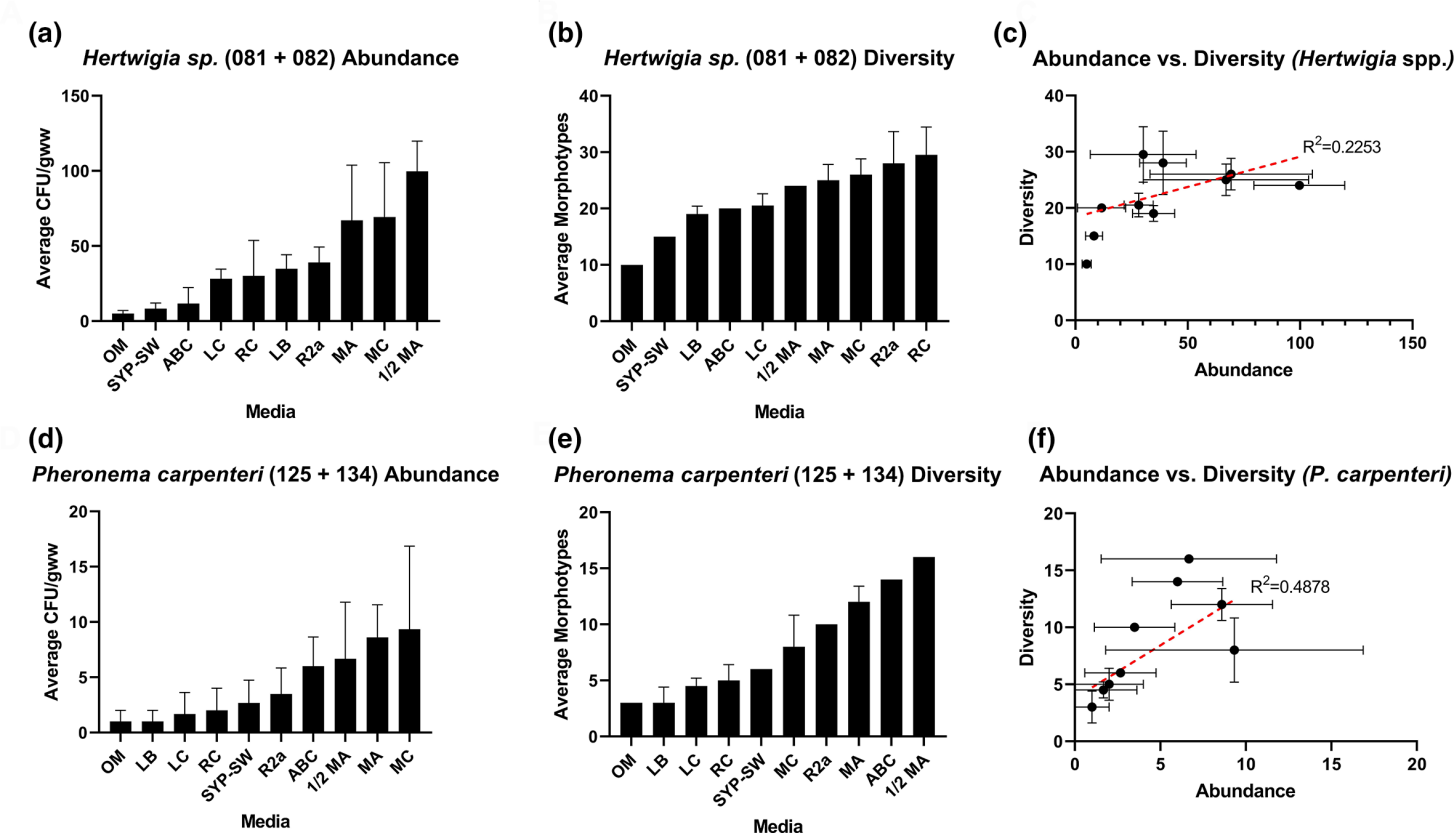
Recovered c.f.u. gww^–1^ counts for isolates obtained from two different samples of two species of hexactinellid sponge. (**a, d**) Abundance counts for bacteria grown across different media for *Hertwigia* sp. and *P. carpenteri*, respectively. Bars: mean+sd. (**b, e**) Number of bacterial morphotypes grown across different media for *Hertwigia*. sp. and *P. carpenteri*, respectively. Media without bars represent counts taken only for GRNL_081. (**c, f**) Linear regression analysis between c.f.u./morphotypes obtained across different media for *Hertwigia* sp. and *P. carpenteri*, respectively (dots represent individual growth media). Bars represent sd. All media were inoculated in triplicate. The number in parentheses in individual graph titles represents sponge replicate ID. Abbreviations: OM, oatmeal agar; SYP-SW, starch-yeast-peptone-seawater agar; ABC, ‘ABC’ agar; LC, LB+carnitine hydrochloride; RC, R2A+carnitine hydrochloride; LB, LB agar; MA, marine agar; MC, marine agar+carnitine.

Preliminary optimization of bacterial culture was carried out for two species of deep-sea hexactinellid sponges using a range of solid growth media. Abundance counts were recorded as counts of individual colonies, irrespective of morphotype, while diversity counts record different morphotypes. The word morphotype here refers to bacterial colonies that were distinguishable using the naked eye. Each colony present on a particular agar was cross-referenced by searching for a colony of the same visible morphology on all other plates of that media type. Parameters including colour, size, border, opacity and profile were taken into account. It should also be noted that these reports of abundance and diversity refer explicitly to cultivability, and not to the overall microbial communities of the sponges. For all media trialled, MA, MC and half-strength marine agar (½ MA) consistently yielded higher colony forming units per gram of wet weight of sponge (c.f.u. gww^–1^) across both sponge samples tested (Fig. 2a–e), while OM produced the lowest (Fig. 2a, d). Of the three, ½ MA was the most successful in recovering bacteria from *Hertwigia* sp., with an average of 99.7 c.f.u. gww^–1^ (Fig. 2a), while MC was the most successful for *P. carpenteri* samples (Fig. 2d), averaging 9.3 c.f.u. gww^–1^. Furthermore, the trend across all media types indicated an approximate 10-fold greater recovery of bacteria from *Hertwigia* sp. samples compared to those from *P. carpenteri*. A significantly higher bacterial abundance was obtained from *Hertwigia* sp. across six of the 10 media used. Media that produced a significantly higher bacterial abundance were LB (*P*=0.0077), RC (*P*=0.0303), R2a (*P*=0.0024), ½ MA (*P*<0.0001), MA (*P*<0.0001) and MC (*P*<0.0001).

RC agar yielded the highest number of bacterial morphotypes for *Hertwigia* sp. (29.5 morphotypes gww^–1^) while ½ MA produced the highest number of bacterial morphotypes for *P. carpenteri* (16 morphotypes gww^–1^) (Fig. 2b, e). The correlation between the number of bacterial isolates and the number of bacterial morphotypes present on each growth medium after 40 days of incubation was quantified. For both sponges, there was a positive correlation between the abundance of bacteria and the diversity as measured by Pearson’s correlation co-efficient (*r*=0.4878, *P. carpenteri*; *r*=0.2253, *Hertwigia* sp (Fig. 2c, f).

### *P. carpenteri* and *Hertwigia* sp. display low overlap in cultivable morphotypes

**Fig. 3.**
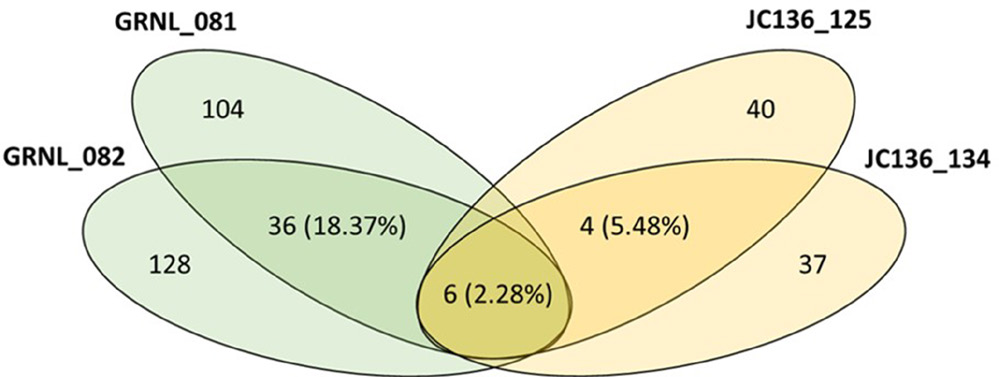
Venn diagram of shared diversity between *Hertwigia* sp. replicates (in green) and *Pheronema carpenteri* replicates (in yellow). The number on the outer edges of the ellipses denotes the number of morphotypes for each individual replicate. The percentage number in the corresponding colour denotes the shared morphotypes between replicates of the same type. The central figure denotes the number of shared morphotypes between all *Hertwigia* sp. and *P. carpenteri* replicates as a whole.

Analysis of the percentage of bacterial morphotypes shared between the two *Hertwigia* sp. biological replicates (GRNL_081 and GRNL_082) (18.37%) was higher than for *P. carpenteri* replicates (JC136_125 and JC136_134) (5.48%). The number of morphotypes that were shared between sponge species was (2.28%) (Fig. 3).

### Change in temperature produces different diversity and abundance of isolates from biological sponge replicates

**Fig. 4.**
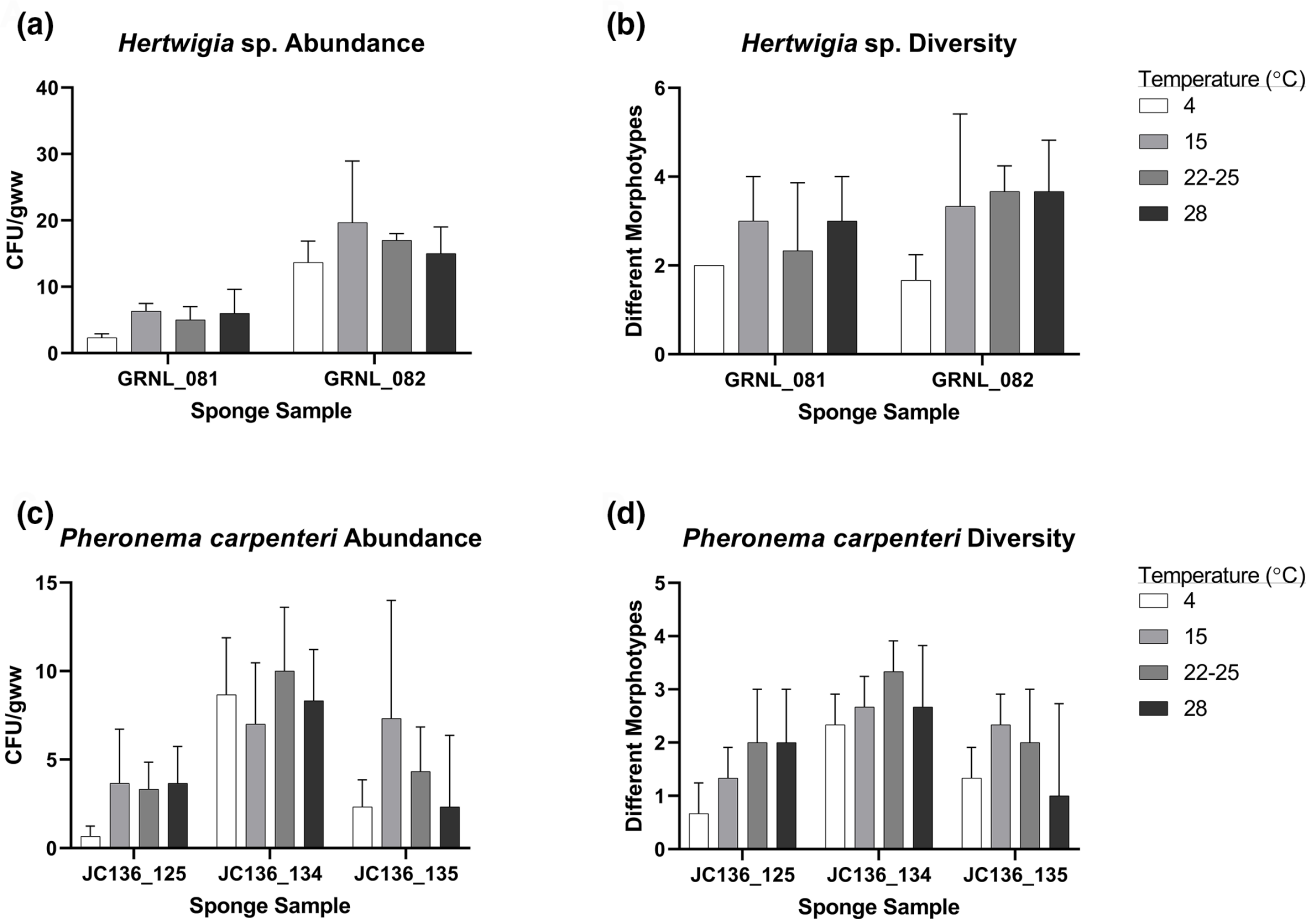
Effect of temperature on the recovery of bacterial isolates across biological replicates of *Hertwigia* sp. and *P. carpenteri*. Effect of temperature on (a) bacterial abundance and (b) diversity in *Hertwigia* sp. Effect of temperature on (c) bacterial abundance and (d) diversity in *P. carpenteri*. All cultures were plated in triplicate. Bars: sd.

The total number of bacteria cultured across all temperatures was greater from *Hertwigia* sp. samples (255 isolates) compared to *P. carpenteri* samples (185 isolates) (Fig. 4a, c), though the difference was not significant [two-way ANOVA; *F*(1.661, 3.322)=0.4792, *P=*0.625]. While intra-species differences were observed, incubating at 15 °C produced the highest number of c.f.u. gww^–1^ in three of five total sponge replicates, while the lowest was observed at 4 °C (Fig. 4a, c). The effect of temperature, however, was not uniform across biological replicates. A two-way ANOVA test examining the effect of sponge individual on isolate recovery showed there was a significant difference between the two *Hertwigia* individuals and isolate recovery [*F*(1,16)=47.28, *P*<0.0001]. Tukey’s post-hoc tests revealed that a greater number of isolates were recovered from GRNL_082 than GRNL_081, and for the temperatures 4 °C (*P*=0.0142), 15 °C (*P*=0.004) and 22–25 °C (*P*=0.009) the two biological replicas were significantly different. The same tests were performed for *P. carpenteri*, showing again that there was an effect of sponge individual on isolate recovery [*F*(2,24)=9.869, *P*=0.0007]. Post-hoc tests showed that at onlyc 4 °C the recovery from sponges JC136_134 and JC136_125 was significantly different.

In terms of bacterial diversity, a higher number of bacterial morphotypes was observed from *Hertwigia* sp. samples at temperatures higher than 4 °C (Fig. 4b). However, the difference in recovery between the 15 °C, 22–25 and 28 °C groups was marginal. For *P. carpenteri*, the highest diversity was seen at the slightly lower temperature of 22–25 °C and the lowest at 4 °C (Fig. 4d).

### Supplementing ½ R2A did not significantly increase diversity or abundance of isolates recovered from *Pheronema* and *Hertwigia* sponges

**Fig. 5.**
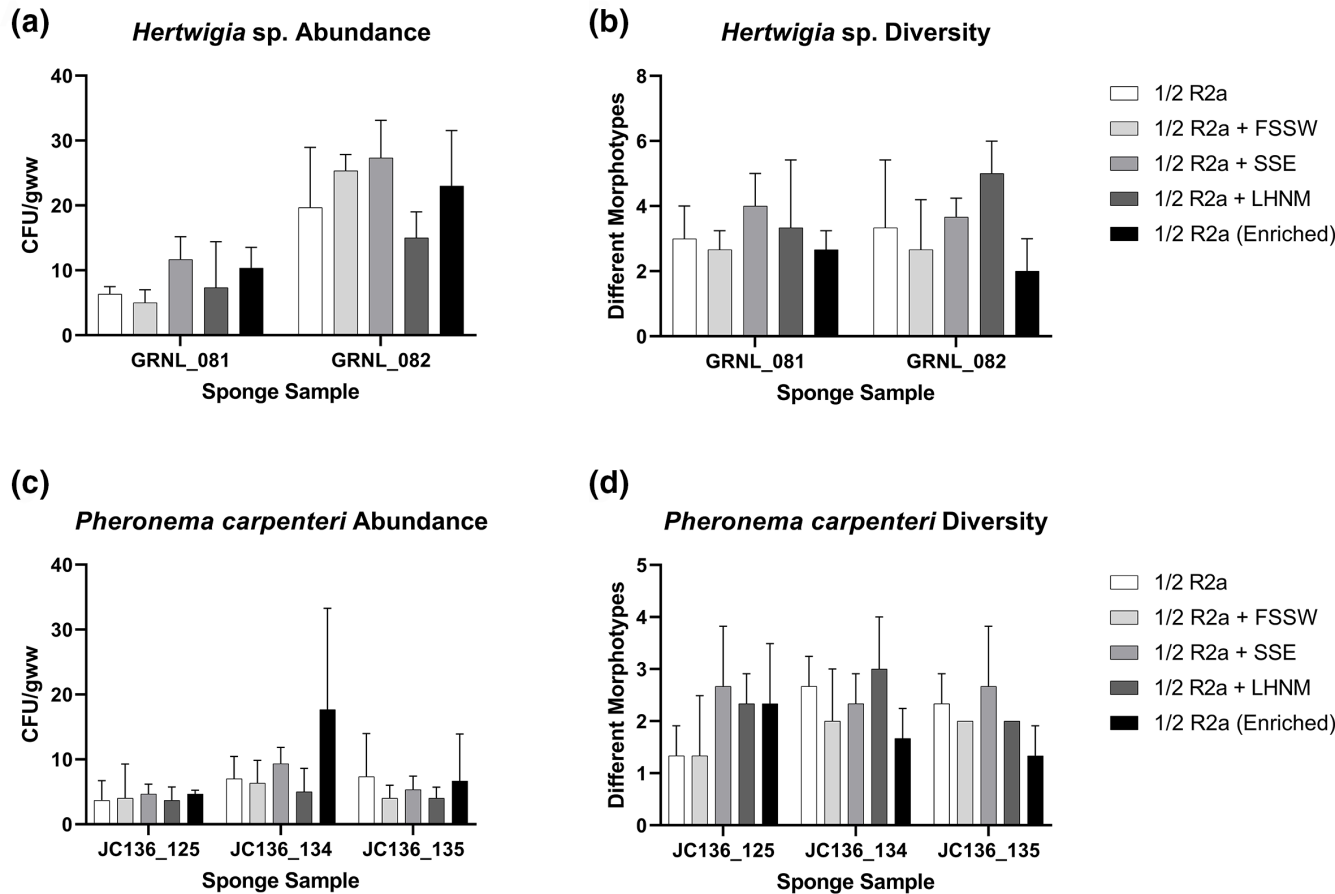
Effect of media supplementation on recovery of bacterial isolates. Effect of supplementation on (a) bacterial abundance and (b) diversity in *Hertwigia* sp. Effect of supplementation on (c) bacterial abundance and (d) diversity in *P. carpenteri*. All cultures were plated in triplicate. Bars: sd. FSSW: filter-sterilized seawater; LNHM: low-nutrient heterotrophic media; SSE: sponge spicule extract.

The effect of media supplementation on bacterial recovery was assessed for both species of sponge, to try and recover increased diversity of cultivable morphotypes. ½ R2A medium was selected as a base medium for supplementation due to its relative success in recovery of isolates displaying antimicrobial activity in preliminary screens (see below). Supplements included either filter sterilized seawater (FSSW), low-nutrient heterotrophic media (LNHM) or SSE, with a fourth treatment including a 24 h enrichment incubation in LNHM prior to plating. The addition of SSE and the inclusion of a 24 h enrichment period produced a higher number of bacterial isolates in both biological replicates of *Hertwigia* sp. (Fig. 5a) and in two of three biological replicates for *P. carpenteri* (Fig. 5c), although it was not statistically significant. The addition of FSSW and LNHM reduced the number of c.f.u. recovered. For both sponges, a higher bacterial diversity was produced overall by the addition of SSE and LNHM. A two-way ANOVA was used to test the effect of media supplements on isolate recovery, revealing a non-significant effect [F(2.304, 23.04)=2.617, *P*=0.088]. Differences were observed between *Hertwigia* sp. individuals [*F*(1,20)=49.64, *P*<0.0001] but not for *P. carpenteri* individuals [*F*(2,30)=3.328, *P*=0.049]. Bacterial diversity was, however, reduced by the addition of FSSW and by the inclusion of an enrichment stage. Again, there was no effect on the number of morphotypes observed following supplementation of ½ R2A [*F*(4,30)=1.845, *P*=0.146].

### Dilution to extinction culture produces more bacterial isolates from *P. carpenteri* than from *Hertwigia* sp

Bacteria were grown from *Hertwigia* sp. (GRNL_081) and *P. carpenteri* (JC136_125) samples using a dilution to extinction (DTE) method [34, 35]. Cell suspensions were diluted to approximately 3–4 cells ml^–1^, inoculated into individual wells of 96-well plates to give an approximate total of ~1 cell per well, and incubated for 40 days.

**Fig. 6.**
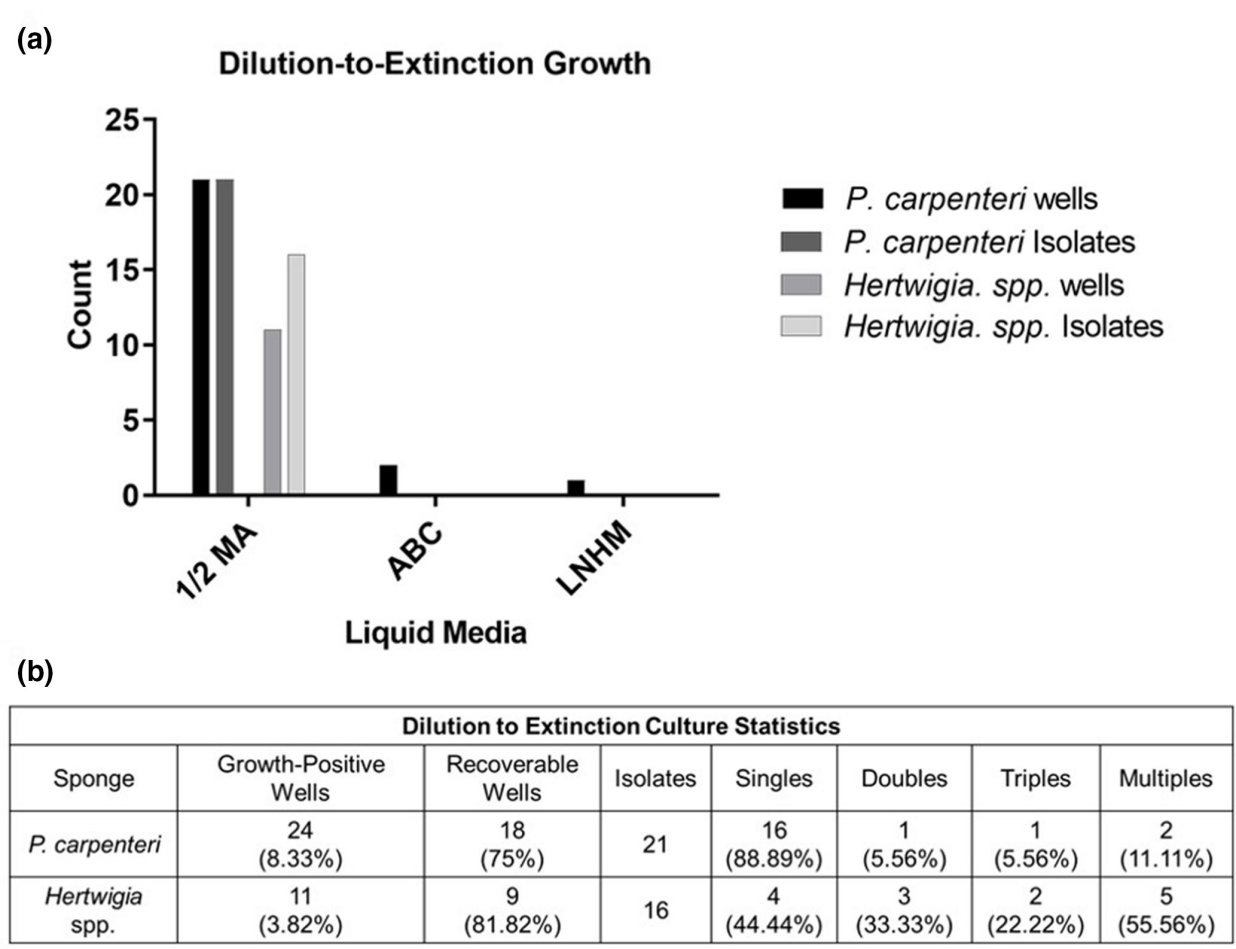
(a) Growth statistics for bacteria grown in different media using the dilution to extinction method. (**b**) Table displaying statistics for the number of bacteria recovered from each treatment. Singles, Doubles and Triples refer to the number of wells from which one, two or three different morphotypes, respectively, were recovered when liquid from wells was plated onto solid media.

For *P. carpenteri*, 21 isolates were recovered on ½ MA, equal to the number of growth-positive wells (Fig. 6a). For *Hertwigia* sp., 16 isolates were recovered, higher than the number of growth-positive wells observed (Fig. 6a). For both sponges, several morphotypes were recovered from some individual wells, indicating mixed cultures (Fig. 6b). No bacteria were recovered on solid media from well-cultures of ABC media or LNHM. A higher number of growth-positive wells, as well as a higher number of bacterial isolates were obtained from *P. carpenteri* (Fig. 6a). A total of 37 bacterial isolates were cultured.

The percentage cultivability of each sponge (based on the Button *et al.* [34] and Connon and Giovannoni [35] methodology), was predicted to be 1.039% for *P. carpenteri* and 0.097% for *Hertwigia* sp. The number of wells containing pure cultures was predicted to be 34.66% for *P. carpenteri*. The actual percentage of wells containing pure cultures, as measured by counting morphotypes cultured on solid media, was 16.67%. The number of wells containing pure cultures was predicted to be 10.78% for *Hertwigia* sp., though the percentage of actual recoverable morphotypes was 4.17%. Cell counting of a bacterial suspension from each sponge using a haemocytometer suggested there to be around twice as many cells present in the *P. carpenteri* suspension, ~2.25×10^7^ cells ml^−1^, compared to ~1.2×10^7^ cells ml^−1^ for *Hertwigia* sp.

### Culturing under altered atmospheric pressure/O_2_ reveals additional bacterial genera from *P. carpenteri*

Further bacterial cultivation was carried out for two separate sponges of *P. carpenteri* (hereafter named Sponge_009 and Sponge_010) collected from a later cruise (Table S3). Further work was carried out on *P. carpenteri* given that a higher number of isolates recovered under a normal atmosphere from this sponge displayed antimicrobial activity than was observed for *Hertwigia* sp. (Table 1, below). Increased pressures were generated with the use of stainless steel chambers (Fig. S5), with the pressure limits determined by the capacity of the chambers used. Gas mixtures were prepared at either 4 or 21 % O_2_ prior to being pumped into the chambers. Sealed chambers were filled with gas mixtures until the desired pressure had been reached. The pressure simulated in this study (5 bar) was equivalent to 4.93 atmospheres, which is representative of an ocean depth of almost 50 m.

**Table 1.** Isolates obtained from hexactinellid sponges that displayed antimicrobial activity in a simultaneous antagonism assay

			Activity vs.		
Parent sponge-Isolate ID	16S rDNA identity based on closest database match	Isolation medium	* M. luteus *	MRSA	* E. coli *
*P. carpenteri*-Ph28	* Bacillus altitudinis *	LB	**+**	**+**	**+**
*P. carpenteri*-RC14	*	RC	**+**	**+**	−
*P. carpenteri*-RC15	*	RC	**+**	**+**	−
*P. carpenteri*-RC17	*	RC	**+**	**+**	−
*P. carpenteri*-A11	* Streptomyces* sp.	RC	**+**	**+**	**+**
*P. carpenteri*-Ph7	* Streptomyces* sp.	½ MA (DTE)	**+**	**+**	−
*P. carpenteri*-NS98	* Micrococcus antarcticus *	R2A	**+**	−	−
*P. carpenteri*-NS10M4	* Bacillus* sp*.*	½ MA	−	−	**+**
*P. carpenteri*-PB091	* Delftia acidovorans *	½ R2A	**+**	**+**	−
*P. carpenteri*-PB109	*	½ R2A	**+**	**+**	−
*P. carpenteri*-PE654	*	½ R2A+SSE	**+**	**+**	−
*P. carpenteri*-PB-104	* Microbacterium paraoxydans *	½ R2A	**+**	**+**	−
*P. carpenteri*-PB-125	* Brevundimonas* sp.	½ R2A	**+**	**+**	**+**
*P. carpenteri*-PC-227	* Microbacterium maritypicum *	½ R2A	**+**	**+**	**+**
*Hertwigia* sp.-RC57	*	RC	**+**	**+**	−
*Hertwigia* sp.-SYP-1	*	SYP	**+**	**+**	−
*Hertwigia* sp.-SYP-2	*	SYP	**+**	**+**	−
*Hertwigia* sp.-RC230	* Delftia acidovorans *	½ R2A	**+**	**+**	**+**
*Hertwigia* sp.-RE707	*	½ R2A+SSE	**+**	**+**	**+**
*Hertwigia* sp.-RI613	*	½ R2A+FSSW	**+**	**+**	−

*M. luteus*: *Micrococcus luteus* (lab strain); MRSA, *Staphylococcus aureus* NCTC 12493; *E. coli, Escherichia coli* NCTC 10418. Isolation media abbreviations as previously described.

*Isolates with low-level activity were not identified using 16S rRNA gene sequencing.

**Fig. 7.**
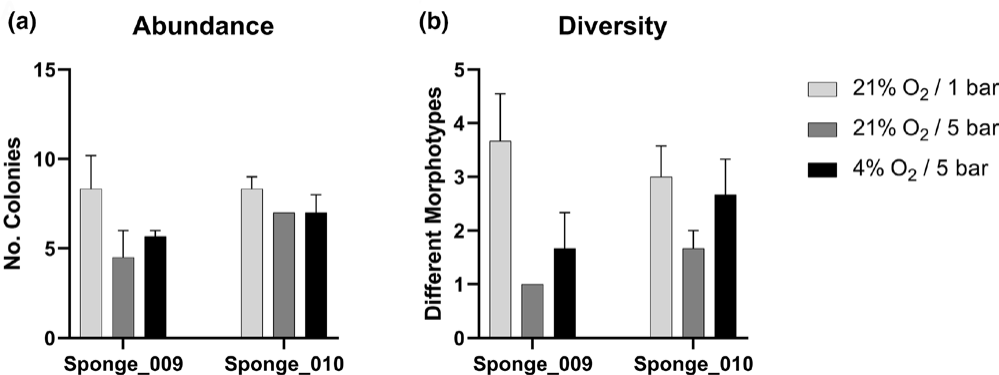
Abundance and diversity measurements for bacteria recovered from *P. carpenteri* and cultured under altered atmospheric conditions. (**a**) Abundance counts. (**b**) Diversity counts. Bars: se across three technical replicates.

Culturing *P. carpenteri* samples at increased pressure (21% O_2_/5 bar) resulted in either a similar or reduced bacterial abundance when compared to those grown under the control conditions (21% O_2_/1 bar) (Fig. 7a). A similar result was also seen for those samples cultured at 5 bar combined with a lower oxygen concentration (4%). A significant reduction in bacterial diversity was also seen for Sponge_009 samples incubated at 21% O_2_/5 bar, when compared to the control group using a two-way ANOVA (*P*=0.0331) (Fig. 7b). A reduction was also observed for those incubated in the lower oxygen concentration, but this was not significant. This indicates that, in these experiments, increased pressure did not improve the abundance or diversity yields from *P. carpenteri* cultured on R2A medium over that of the control conditions.

Bacteria from each environmental condition were subcultured onto solid media in non-pressured conditions and genomic DNA was extracted from each individual morphotype cultured. Sequencing, quality-trimming and aligning of the forward and reverse 16S rRNA gene sequence resulted in data for 39 isolates, as shown in the histograms for sequence length, query cover (%) and identical sites (%) of all blastn queries (Figs S1–S3).

The top blastn hits for all 16S rDNA sequences were filtered to those that matched with 100% query cover and a <97% site identity. Filtering sequences in this way revealed the presence of a single 16S rDNA sequence that may represent a potentially novel *Bacillus* species. The sequence was from an isolate recovered at 21% O_2_/atmospheric pressure and matched most closely to *Bacillus* sp*.* ITP29 with an identical site percentage of 96.6%. The filtering cutoffs were relaxed to also include those that had an identical site percentage of <99%, in accordance with updated operational taxonomic unit (OTU) clustering recommendations [36]. By including all 16S rDNA sequences that were divergent from those in the NCBI database by >1%, rather than by >3%, the number in the filtered list increased from one to nine, as only one sequence had an identical site match of <97%. The nine 16S rDNA sequences that had an identical site match of <99% matched to sequences related to *Psychrobacter piscatorii *, *Pseudomonas* sp*.*, *Erythrobacter* sp*.*, *Dermacoccus nishinomiyaensis *, *Bacillus* sp*.* and ‘Uncultured bacterium clone Md-9.

Of the 39 isolates, 15 were obtained from Sponge_009, whereas 24 were obtained from Sponge_010. All 15 sequences from Sponge_009 had unique blastn identities, 13 of which (86.7%) were unique to Sponge_009. A total of 18 of the 24 sequences from Sponge_010 had unique blastn identities, 16 of which (88.9%) were unique to Sponge_010. Two sequences from each sponge had a shared blastn identity. Of the 31 unique blastn identities, 29 (93.5 %) of these were unique to a particular sponge.

**Fig. 8.**
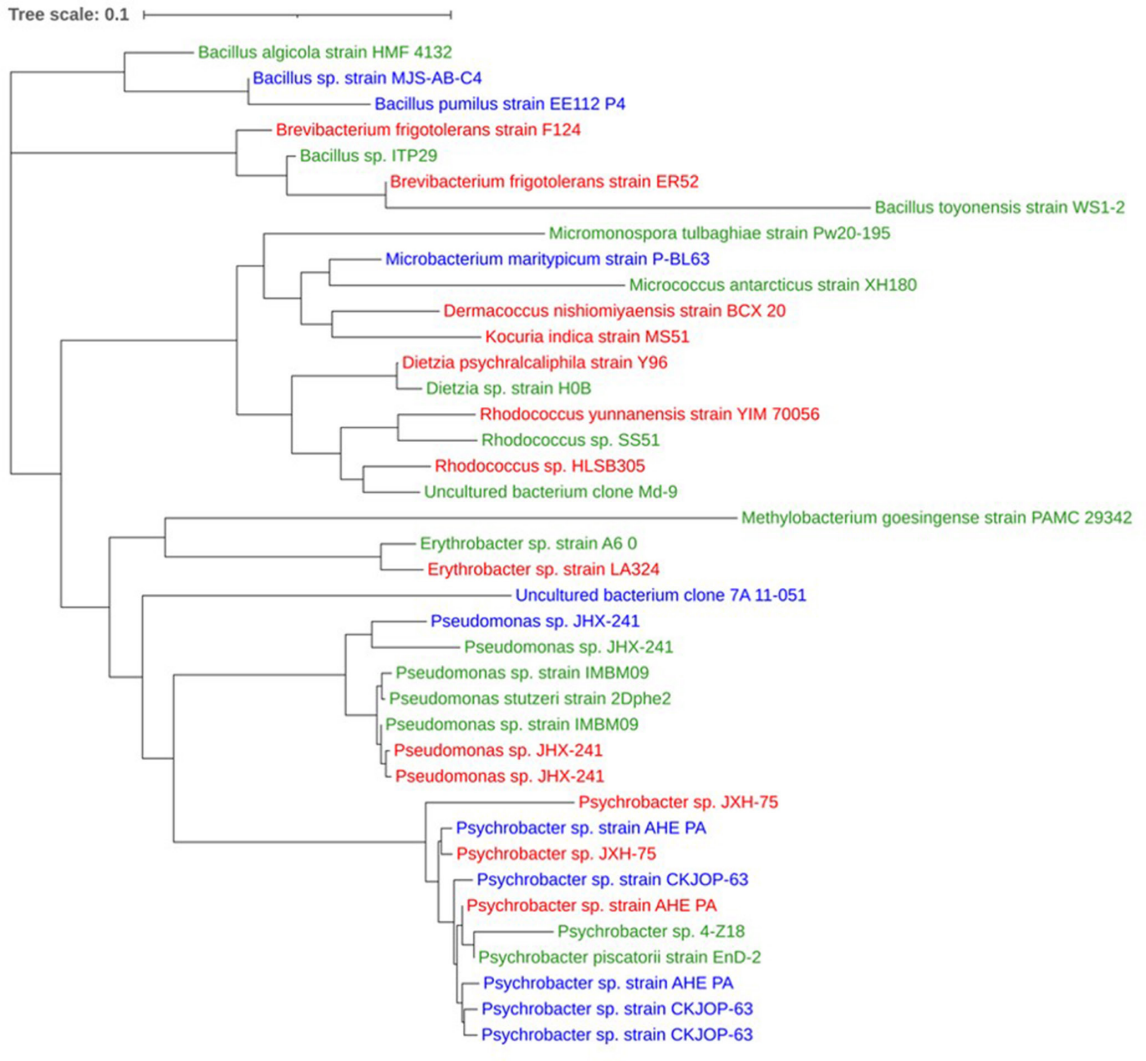
Neighbour-joining tree of 16S rDNA sequences obtained from bacterial isolates cultured from *P. carpenteri*. Taxonomic IDs represent the top blastn hit for each sequence. Colours represent the treatment at which each isolate was recovered. Green: 21% O_2_/1.01 bar; blue: 21% O_2_/5 bar; red: 4% O_2_/5 bar.

A neighbour-joining tree of all 39 16S rDNA sequences (alignment length 1621 nt) from each sample treatment revealed that they did not cluster together at the species level, indicating that isolates from each sample treatment were not more closely related (Fig. 8).

**Fig. 9.**
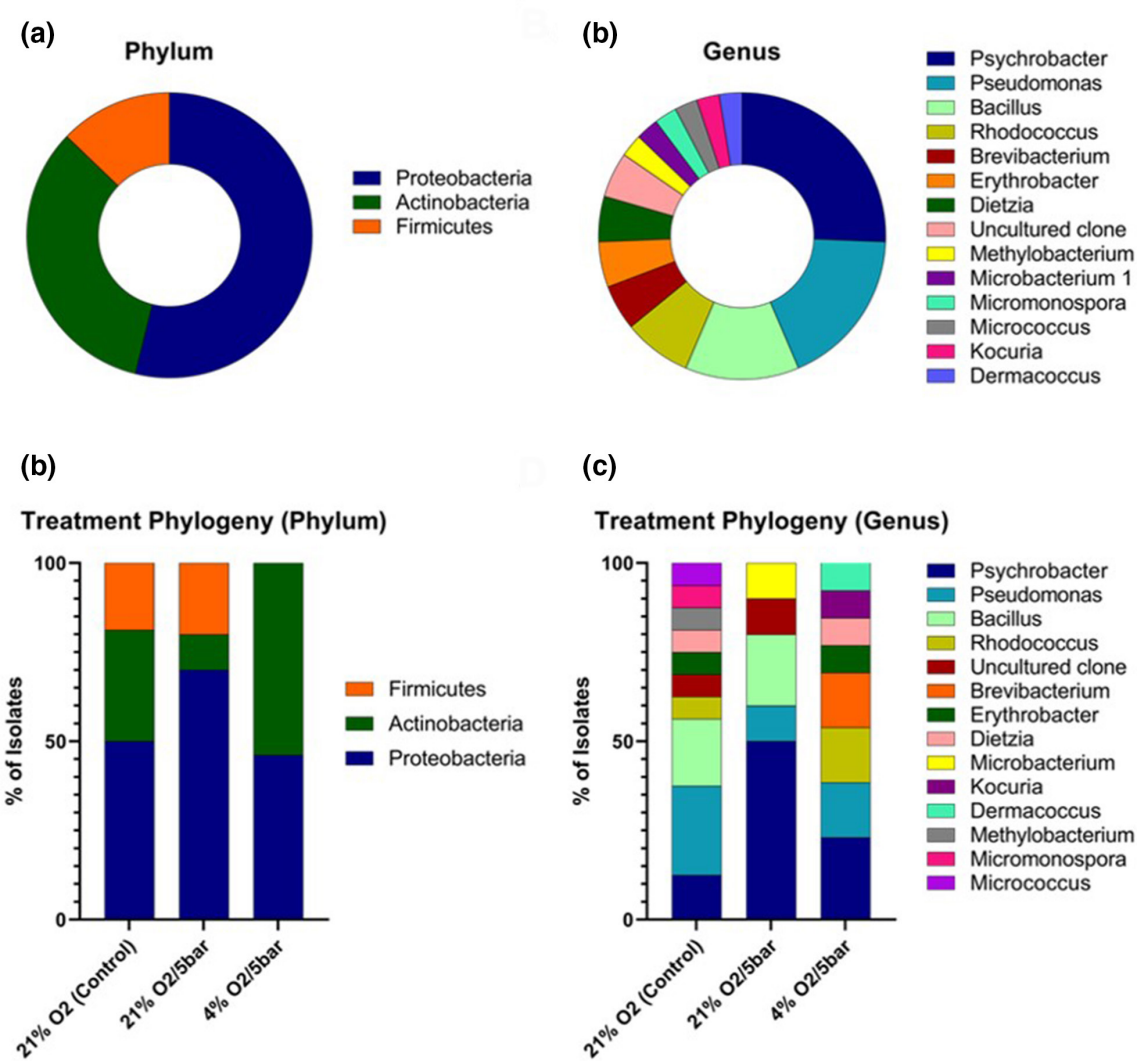
(a) Phylum- and (b) genus-level distribution of all *P. carpenteri* isolates sequenced. (**c**) Phylum- and (d) genus-level distribution of isolates recovered, split by sample treatment.

A total of 39 different morphotypes were recovered from the *P. carpenteri* samples, belonging to 13 genera (Fig. 9a) within the phyla *Proteobacteria *, *Actinobacteria* and *Firmicutes* (Fig. 9b). Of these, 16 (41%) were from 21% O_2_/1.01 bar, ten (25.6%) from 21% O_2_/5 bar, and 13 (33.3%) from 4% O_2_/5 bar. The most common genus recovered was *Psychrobacter* (25.6%), a proteobacterium present in all three sample types (Fig. 9c). Bacteria from all three phyla were recovered from 21% O_2_/1.01 bar and 21% O_2_/5 bar, but no *Firmicutes* were identified in the 4 % O_2_/5 bar (Fig. 9d).

While increased pressure reduced overall bacterial abundance, it appeared to promote the recovery of certain genera that were not cultured under the control conditions. An isolate of the genus *Microbacterium* genus was found in the samples incubated at 21% O_2_/5 bar but not at 21% O_2_/1.01 bar or at 4% O_2_/5 bar, indicating that a combination of higher oxygen and pressure may favour the recovery of this specific genus. *Dermacoccus *, *Kocuria* and *Brevibacterium* were only cultured on R2A at 4 % O_2_/5 bar, while *Micrococcus, Micromonospora* and *Methylobacterium* were only cultured at 21 % O_2_/1.01 bar (Fig. 9d), further evidencing the need to implement different environmental factors when attempting to improve recovery of sponge bacterial diversity.

### Bacteria from hexactinellid sponges display antimicrobial activity against clinically relevant bacterial strains

A total of 1115 bacterial isolates were screened for antimicrobial activity using a simultaneous antagonism assay. Briefly, a confluent lawn of an indicator organism was spread across an agar plate and ‘spotted’ with sponge isolates. The plates were incubated and assessed visually for a ‘zone of inhibition’ around each sponge isolate, with a total of 621 (55.7%) isolates from *Hertwigia* sp. and 494 (44.3%) from *P. carpenteri*. The potential screening of duplicate strains was a possibility given isolates were obtained at different stages and via different methods. Each isolate was initially screened for antimicrobial activity against the three test bacteria *M. luteus *, *S. aureus* and *E. coli* (Table 1). *S. aureus* and *E. coli* were chosen for their clinical relevance as well as to represent both Gram-positive and Gram-negative bacteria. *M. luteus* in particular was chosen due to its relative susceptibility to antimicrobials, thought to be a reflection of its small genome and resulting lack of certain resistance proteins found in other *Actinobacteria* [37]. *M. luteus* was therefore considered a good target, or ‘wide net’, for first-pass antimicrobial detection. Of the 20 isolates that displayed antimicrobial activity, 18 (90%) were active against both Gram-positive organisms, while the 19^th^ isolate, *Micrococcus antarcticus *, showed activity against *M. luteus* only. Six isolates (30%) were active against both Gram-positive and Gram-negative organisms, with those that were identified coming from all three phyla recovered (* Actinobacteria *, *Proteobacteria* and *Firmicutes *), while only one isolate (5%) was solely active against *E. coli *.

## Discussion

### Culture using solid media and community dissimilarity

Most studies investigating bacteria with antimicrobial activity from sponge microenvironments have focused on those sponges collected from shallow waters (<200 m deep) and those from the class Demospongiae. To optimize the culture of bacteria from two previously uncharacterized species of hexactinellid sponge, bacteria were cultured on a range of solid-growth media, representing the first culture-based microbial analysis of these two sponges. The number of c.f.u. and the number of distinct morphotypes (i.e. abundance and diversity) were also compared between isolates obtained from *P. carpenteri* and *Hertwigia* sp. In general, this revealed a higher abundance of isolates from *Hertwigia* sp. and the positive impact of lower-nutrient media designed to mimic the marine environment, across both sponge species. The inclusion of seawater in culture media (SYP-SW), whilst resulting in low bacterial abundance, did produce a relatively high number of bacterial morphotypes, particularly in the case of *Hertwigia*. The use of carnitine, a substrate for which sponge bacteria have previously been shown to display metabolic specialization [38], did not appear to significantly increase the number of bacterial c.f.u. or morphotypes.

Maximizing the chance that recovered bacterial isolates are derived from the marine/sponge environment attempts to reduce the chance for re-discovery of previously characterized antimicrobials by taking advantage of the increased species and functional richness associated with deeper waters [39, 40] and the number of marine natural products that have been discovered from the marine environment in recent years [7, 8]. It is also important for studies seeking to screen recovered isolates for antimicrobial activity to consider which media produce the highest number of distinct bacterial morphotypes (i.e. diversity), rather than just abundance – a higher number of individual morphotypes provides a higher number that can be screened. It should be noted that whilst two isolates display similar morphologies, this does not mean that they will produce the same profile of secondary metabolites. Distinguishing colonies based on visual morphology attempts to provide an early, simple de-replication step in the process of identifying potentially novel antimicrobial candidates. However, this is limited by the fact that strains may display different morphologies when using different culture media, and that different bacteria may display highly similar morphologies that may be indistinguishable with the naked eye.

These observations provide an initial reassurance that the bacteria cultured are more likely to be those present in the marine environment, rather than the result of terrestrial contamination. Whether the bacteria cultured are truly ‘sponge-associated’ as opposed to being just from the marine environment in general was not investigated, and is unknowable without further investigation of metabolic and/or genetic specialization. This indication was further explored by the molecular characterization of isolates obtained from culture at altered atmospheric pressure, as will be discussed later.

The use of solid growth media resulted in a ten-fold increase in the number of colonies recovered from *Hertwigia* sp., although a similar increase was not observed for diversity, suggesting the *Hertwigia* sp. microbiota is dominated by either one or a small number of bacterial species that cannot be cultured. Cell counting techniques used in DTE experiments indicated there to be around twice as many cells present in the *P. carpenteri* suspension. Whilst cell counting techniques using only a haemocytometer are rudimentary, it does perhaps suggest the *P. carpenteri* sponge not only has a higher bacterial density but also a higher number of constituents that were either ‘uncultivable’ under the conditions applied, or perhaps unviable. Use of DTE experiments indicated that *Hertwigia* species have a much lower microbiome cultivability (0.097%) than *P. carpenteri* (1.039%). The formula for calculating percentage cultivability of bacteria devised by Button *et al.* [34] has since been updated to include information concerning the relative abundance of particular species within bacterial suspensions [14]. Culture of bacteria here by DTE would benefit from a 16S rRNA taxonomic survey of the host microbiota, in order to provide this information.

Another initial indication of the community dissimilarity of each sponge species is the markedly low overlap between shared morphotypes [six (2.26%) of 269 isolates]. Whilst this observation is limited by the small number of isolates obtained from *P. carpenteri*, it is supported by the fact that 87% of the 16S rDNA sequences obtained from *P. carpenteri* had blastn identities that were found only a particular sponge replicate. It also appears that the different media used herein revealed distinct groups of bacterial morphotypes. This was noted in visual observations and is also highlighted by the lack in correlation between an increase in abundance and diversity. The increase in number of bacterial colonies recovered did not lead to a significant increase in a specific morphotype, with the increase being made up of representatives of numerous morphotypes.

### Comparison between biological replicates

Due to the lack of information available on the hexactinellid microbiota, it is unknown how much variation exists between biological replicates of the same species. Perhaps the most extensive overview of the sponge microbiota to date included fewer than five biological replicates for the majority of species analysed [41]. It is interesting to note than even for species for which a high number of biological replicates was provided, there was still a high degree of variation in community dissimilarity as determined by 16S rDNA sequence profiling [41]. In our efforts to observe the effects of isolation media, temperature and supplementation on culturing effects, we observed significant differences between biological samples belonging to the same species. In this study, sponge samples were classified as biological replicates if belonging to the same species and if they were retrieved from the same sampling event and depth. This attempts to control the effect that depth, a proxy for temperature and pressure, may have on the recoverability of sponge-associated bacteria. The *P. carpenteri* sponges JC136_125 and 136 were both sampled from the same location at 1051 m depth, whereas the two *Hertwigia* sp. sponges were sampled at 2175 m (GRNL_82) and 2227 m (GRNL_81) depth (Fig. S3).

The most comprehensive analysis of the hexactinellid microbiota to date included three or four replicates of each species from 770 to 4160 m depth [22], reporting that the relative abundance of each taxon varied more widely between biological replicates of hexactinellid sponges than for demosponges. Members of the genera *Hertwigia* and *Pheronema* were absent from the Steinert study. Using the amplicon sequence variant (ASV) technique of classification [42], a greater dissimilarity in alpha-diversity was also observed between 33 replicates of the hexactinellid *Vazella pourtalesii*, compared to the microbiome of surrounding seawater and sediment, at ASV and phylum level [24]. It is therefore relevant to consider the differences in community composition between biological replicates of the same host species, particularly for those that have not been previously investigated. It appears from the preliminary investigation conducted here that cultivable differences are more pronounced between sponge species than for replicates of the same sponge species. Further in-depth 16S rRNA gene or metagenomic community profiling would be recommended to fully explore these inter- and intra-sponge differences.

### Dilution-to-extinction culture

Bacteria from *Hertwigia* sp. and *P. carpenteri* were cultivated using the DTE method. Whilst the use of ½ MA produced colonies in 11/96 and 24/96 wells for *Hertwigia* sp. and *P. carpenteri*, respectively, the use of ABC and LNHM produced only a small number of colonies for *P. carpenteri* and none for *Hertwigia* sp. LNHM [43] and sterilized seawater [35] have previously been used for the cultivation of isolates from marine water. Given the success of ABC media in improving the diversity of bacteria recovered on solid media, it is surprising that such a low number were produced using DTE in the current study. A theoretical outcome of using DTE is that wells are more likely to contain members of the most highly abundant species present in the original sample. The observation that a lower number of isolates were obtained from *Hertwigia* sp. may support the concept that the microbiota is dominated by one or several ‘uncultivable’ species. However, this does not provide an explanation for why such a low number of isolates were produced from *P. carpenteri* when using ABC and LNHM. The calculated cultivability value was much lower for *Hertwigia* sp.

### Culture at increased atmospheric pressure

Bacteria from the phyla *Proteobacteria *, *Actinobacteria* and *Firmicutes* were recovered from *P. carpenteri* samples cultivated under increased atmospheric pressure, at 21% O_2_/5 bar and at 21% O_2_/1 bar. Interestingly, the use of 4% O_2_/5 bar prevented the growth of *Firmicutes *. The addition of pressure to culture conditions appeared to increase the overall percentage of *Proteobacteria* isolates. The control group (21% O_2_) resulted in the highest abundance and diversity recovered, while lowering the O_2_ to 4% resulted in no *Firmicutes* being recovered but increased the percentage of *Actinobacteria* isolates. Bacteria cultured under the same atmospheric pressure/O_2_ did not appear to be more closely related, or cluster together in terms of 16S rRNA gene similarity. There were, however, species that were unique to each atmospheric condition.

The addition of pressure (21% O/5 bar) reduced the overall number of bacteria cultured, but produced isolates belonging to *Microbacterium* and an ‘uncultured bacterial clone’. Isolates grown under a low-O_2_, pressurized environment (4% O_2_/5 bar) included members of *Dermacoccus *, *Kocuria* and *Brevibacterium *.

The lack of data from 4% O_2_ at atmospheric pressure in this study prevents a more comprehensive analysis into whether certain isolates were selectively cultured by the combination of low O_2_ and pressure. The ability of all isolates to grow in non-pressurized environments once sub-cultured, however, demonstrates that none of them require pressure for their growth. It may also be that the combination of pressure and low O_2_ concentration prevented the growth of competing bacteria in the first instance. Isolates that did not require pressure for their growth may also have grown more favourably under pressurized conditions. Isolates belonging to the psychrotolerant *Psychrobacter* [44] were the most common across all sample treatments.

The sponges used in this study were obtained from depths ranging between 1051 and 2228 m, which is equivalent to ~105–223 atmospheres of pressure. The pressure simulated in this study (5 bar) was equivalent to 4.93 atmospheres, which is representative of a depth of almost 50 m. Previous work detailing the range of cellular processes affected by pressure suggest that essential biological processes (in *E. coli *) are not prevented until pressures much higher than this [45]. Therefore, it is assumed that many bacterial species associated with *P. carpenteri* and *Hertwigia* sp. may be cultivable at much higher pressures than those used in this study. Further work in assessing the impact of higher pressure on the cultivation of sponge-associated bacteria, and their ability to thrive and survive at such pressures would be of benefit in exploring this, though the practicalities of doing such work may represent a significant hurdle. The pressures at which bacteria were grown here were determined by the pressure capacity of the containers used.

### Antimicrobial screening

A total of 20 (1.79%) of the 1115 isolates screened displayed antimicrobial activity. Previous reports of antimicrobial screening vary with respect to sample and methodology. Previous studies from sponges have reported hit rates of 8.4–41% without dereplication strategies [46–48]. Previous studies in soil and wastewater have reported hit rates of 1.3–42.4% [49, 50]. High-throughput studies screening small-molecule libraries report a hit rate of ~0.5% [51]. Of the 20 isolates that displayed antimicrobial activity, 16 were recovered from *P. carpenteri*. This is perhaps surprising considering the much lower number of isolates obtained from this sponge, compared to the *Hertwigia* sp. samples. The prevalence of active isolates was used to select a sponge for bacterial culture at increased pressure, but it remains to be seen whether the *P. carpenteri* microbiota contains more bacteria that produce antimicrobial compounds *in vitro*. Observations about the potential composition of the *P. carpenteri* microbiota provided by DTE culture may become relevant if bioactive bacteria cultivated from *P. carpenteri* are found to include members of the rare biosphere. The majority of isolates that displayed antimicrobial activity did so against Gram-positive organisms, while several were also active against *E. coli* NCTC10418. The need for the discovery of novel antimicrobial agents active against Gram-negative organisms is of particular importance, given the lack of available agents effective against drug-resistant members of this group [52]. Isolates that appeared to display inhibitory activity towards *E. coli* NCTC10418 were recovered from both sponge species studied here, indicating the value in continued investigation of the bioactive potential of their associated microbiota.

## Conclusions

An evaluation of bacterial culture-based methods was carried out for two previously uncharacterized species of the hexactinellid sponge, revealing a higher number of cultivable isolates from *Hertwigia* sp. and a higher proportion of bioactive isolates from *P. carpenteri*. The use of elevated atmospheric pressure was demonstrated to have an impact on the bacterial genera that were capable of being recovered. Isolates were screened for antimicrobial activity, producing several isolates of interest, active against Gram-positive and Gram-negative bacteria. These have been prioritized for downstream analysis. This study constitutes the first exploration of the diversity and antimicrobial potential of the microbiota from *P. carpenteri* and *Hertwigia* sp. sponges, as well as the use of pressure in culturing bacteria from such samples. It appears that the cultivation of isolates with antimicrobial potential from *P. carpenteri* is more likely than from *Hertwigia* sp. However, there also exists intra-species dissimilarity between cultivable bacteria from both sponges. Further molecular, microbiome-level investigation would be recommended to examine trends in detail. Overall, the isolates obtained herein provide a promising avenue for further investigation and indicate that *Pheronema* sponges are promising targets for the isolation of novel antimicrobial candidates. The 16S rRNA gene sequences generated by this study were submitted to GenBank under accession numbers MZ723441 to MZ723479. A full list of accession numbers can be found in Table S4.

## Supplementary Data

Supplementary material 1
