# Supplementary material for: Impact of growth media and pressure on the diversity and antimicrobial activity of isolates from two species of hexactinellid sponge
Source: Microbiology (Reading). 2021 Dec 13;167(12):001123. doi: 10.1099/mic.0.001123 (PMC8744994; doi:10.1099/mic.0.001123)
Supplement: Supplementary material 1 [file mic-167-1123-s001.pdf]

Supplementary Data S1

Table 1 – Media used for the culture of bacterial strains I this study

Media/Component	Code	Composition	Reference
**ABC (Agar)**	ABC	25g/L MgSO4·7H2O, 22.5g/L (NH4)2SO4, 7.5g/L Na2SO4·10H2O, 2.5g/L KH2PO4, 2.5g/L KCl, 0.7g/L Ca(NO3)2·4H2O, phosphate buffer (20mM final), 0.1g/L peptone, 0.1g/L yeast extract, 0.1 g/L glucose, ddH_2O, 1.5% agar (Sigma-Aldrich)	Kato et al., 2018
**LB, Miller**	LB/L	1.5% agar (Sigma-Aldrich), ddH_2O	-
**LB, Miller + Carnitine**	LC	1.5% agar (Sigma-Aldrich), ddH_2O, 0.2g/L Carnitine Hydrochloride	-
**Low nutrient heterotrophic media**	LNHM	1.0 μM NH_4Cl, 0.1 μM KH_2PO_4, and vitamin mix[(a)] at a 10^{-4} dilution of stock 1.5% agar (Sigma-Aldrich), ddH_2O	Cho and Giovannoni, 2004
**Marine Agar**	MA/M	37.4 g/L Marine Broth 2216 (BD Difco), 1.5% agar (Sigma-Aldrich), ddH_2O	-
**Marine Agar + Carnitine**	MC	37.4 g/L Marine Broth 2216 (BD Difco), 1.5% agar (Sigma-Aldrich), ddH_2O, 0.2g/L Carnitine Hydrochloride	-
**Marine Broth**	MB	37.4 g/L Marine Broth 2216 (BD Difco), ddH_2O	-
**Oatmeal Agar**	OM	72.5 g/L Oatmeal agar (Sigma), 33.3 g/L Instant Ocean™, ddH_2O	-
**ABC, Broth**	ABC	Basal salts, 1.5% agar (Sigma-Aldrich), phosphate buffer (20mM final), 0.1g/L peptone, 0.1g/L yeast extract , 0.1g/L glucose	Kato et al., 2018
**R2a + Carnitine**	RC	18.1 g/L R2a, 1.5% agar (Sigma-Aldrich), ddH_2O, 0.2g/L Carnitine Hydrochloride	-
**R2a + Filter Sterilised Seawater**	FSSW	9.05 g/L R2a, 0.22-µm filter sterilised seawater	
**R2a + Sponge spicule extract**	R2a + SSE	9.05 g/L R2a, 40 ml/L sponge spicule extract[(b)], 1.5% agar, 33.3 g/L Instant Ocean™, ddH2O	Sipkema et al, 2011; Keren, Lavy and Ilan 2016;
**Reasoner's 2A agar**	R2a/R	18.1 g/L Reasoner’s 2 agar (Oxoid), 33.3 g/L Instant Ocean™, ddH_2O	-

Media/Component	Code	Composition	Reference
**R2a + 24 h LNHM Enrichment**	R2a + 24 h Enrich.	10 g sponge homogenate was centrifuge (4696 x *g*, 20 min) and pellet re-suspended in 10 ml LNHM and incubated at 4°C for 24 h. Enriched inoculated was inoculated on ½ R2a.	Bowman 2001; Cho and Giovannoni 2004
**Starch-Yeast-Peptone-Seawater**	SYP-SW	10g/L Starch, 4g/L Yeast Extract, 2g/L Peptone, Autoclaved Seawater	Margassery et al., 2012
**Tryptic Soy Agar**	TSA	1.5% agar (Sigma-Aldrich), ddH_2O	

[a] Thiamine HCl 0.2 mg/L, biotin 1 µg/L, vitamin B_{12} 1 µg/L, Folic acid 2 µg/L, Pabs 10 µg/L, Nicotinic acid 0.1 mg/L, Inisotol 1.0 mg/L, Calcium panthothenate 0.2 mg/L, Pyradoxine HCl 0.1 mg/L

[b] 10 g sponge host tissue were homogenised with sterilised mortar and pestle. Homogenate was extracted overnight in 50 ml ddH_2O and filtered sterilised using a 0.22-µm filter. Cake remaining on filter was suspended in 50 ml ddH_2O, centrifuged at 138 x *g* for 10 min and pellet re-suspended in 20 ml ddH_2O to make SSE (Sipkema *et al.* 2011).

24 h enrichment

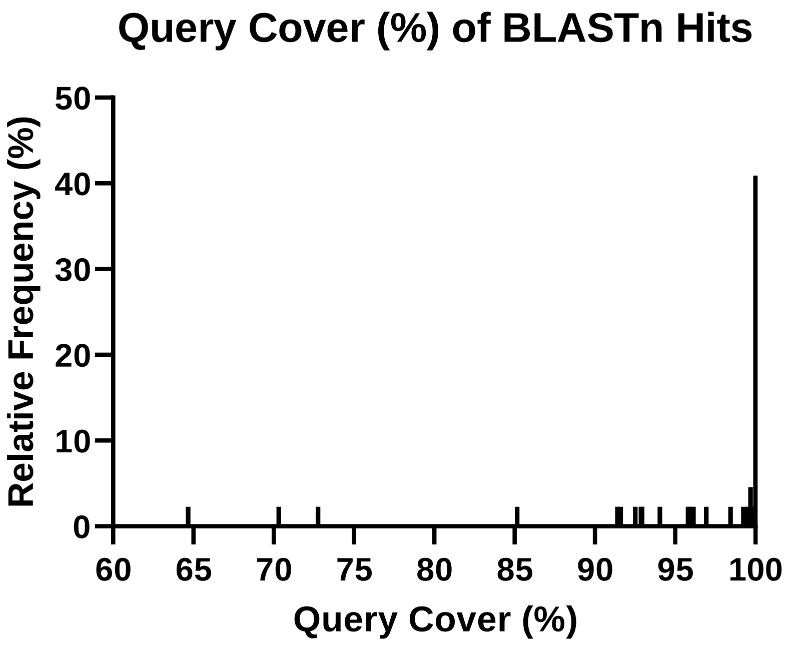

Figure S1 – Query Cover (%) matches for 16S rDNA sequences submitted to BLASTn

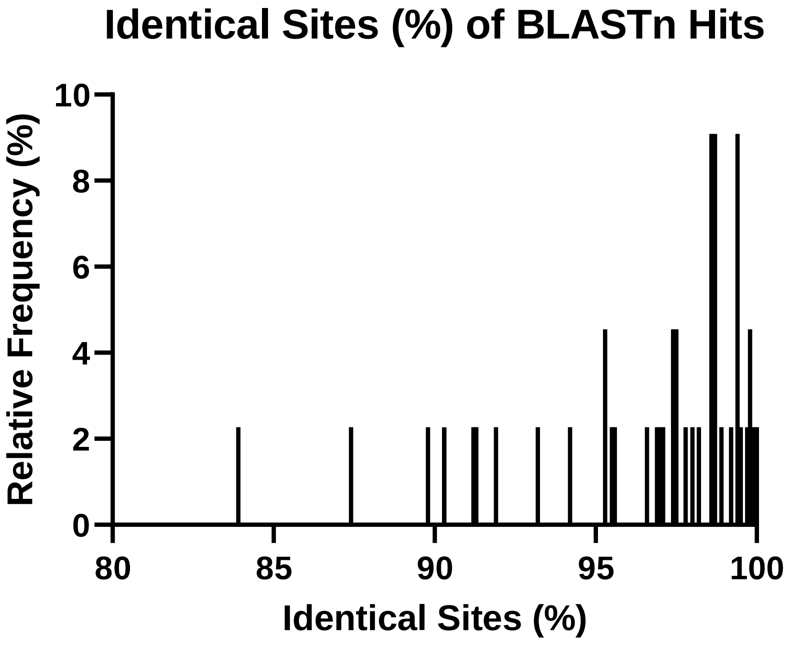

Figure S2 – Identical site (%) matches for 16S rDNA sequences submitted to BLASTn

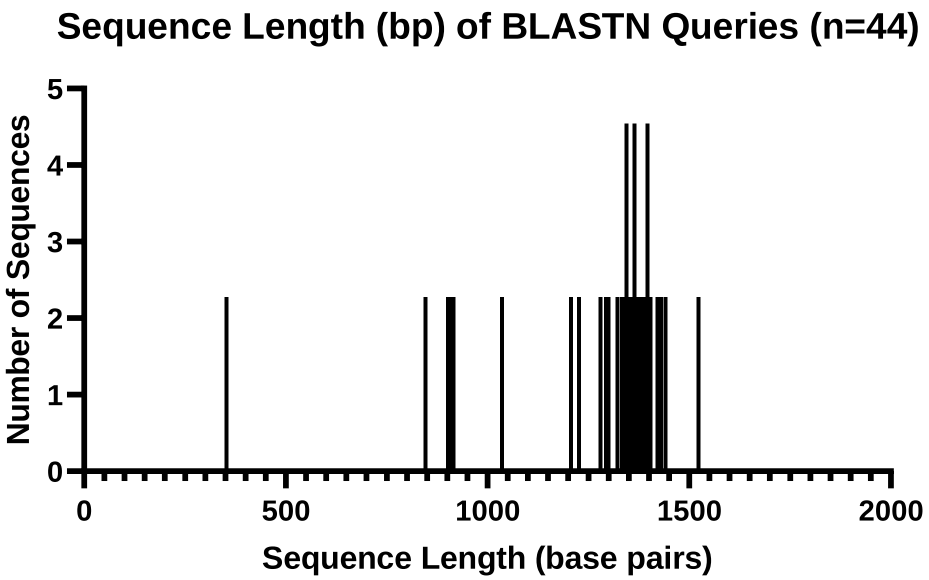

Figure S3 – Sequence length (base pairs) of 16S sequences submitted to BLASTn

Table 2 – Community richness and diversity estimates for isolates cultured under different atmospheric conditions

	Chao1 Richness	Shannon Diversity	Simpson Diversity	Pielou Evenness
21% O_2/ 1.01 bar	37	2.625048	0.919668	0.96935
21% O_2/5 bar	22	1.666333	0.743802	0.856326
4% O_2/5 bar	16.5	2.069202	0.857143	0.941734

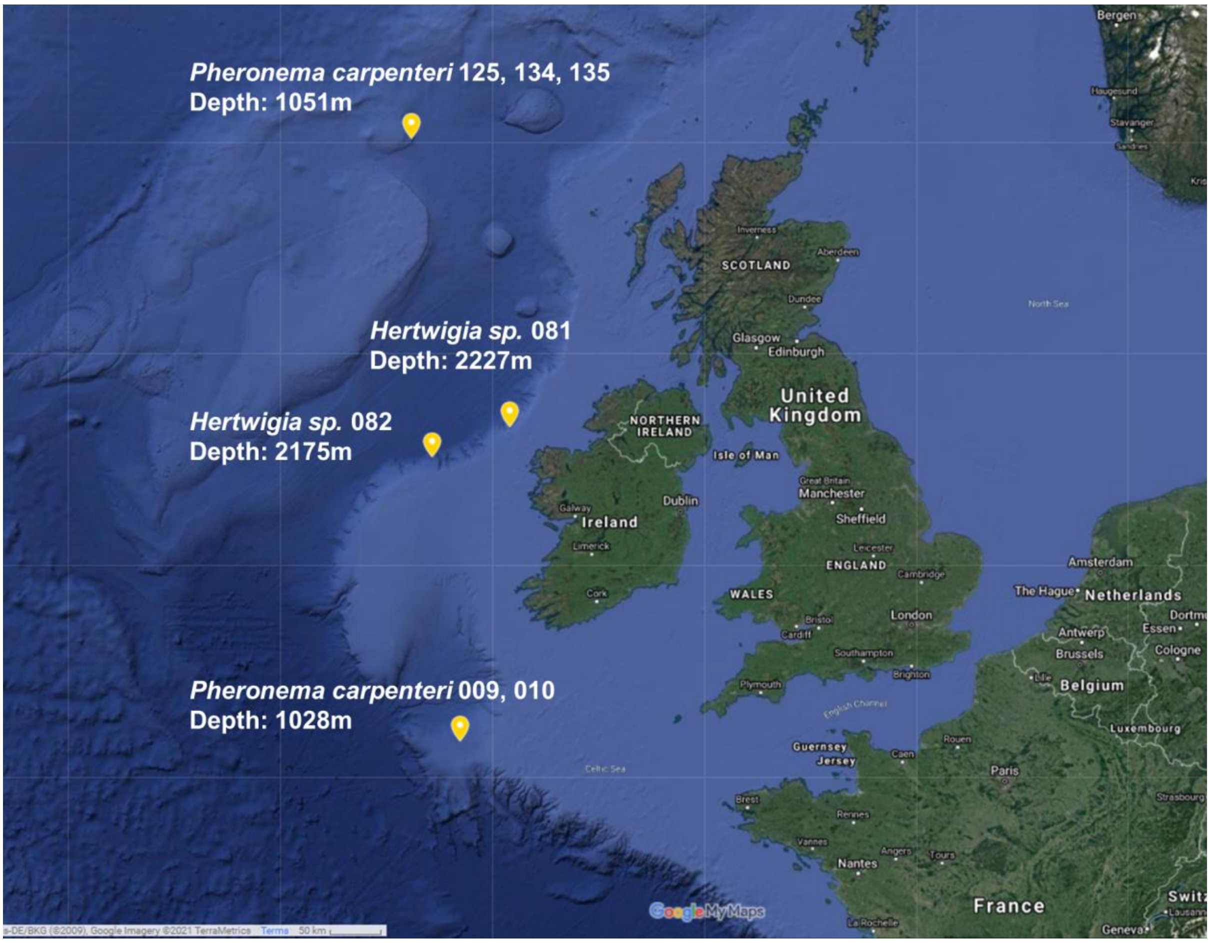

Figure S4 – GPS co-ordinates of collection sites for sponges used in this study. Picture obtained by inserting sample co-ordinates into Google Maps.

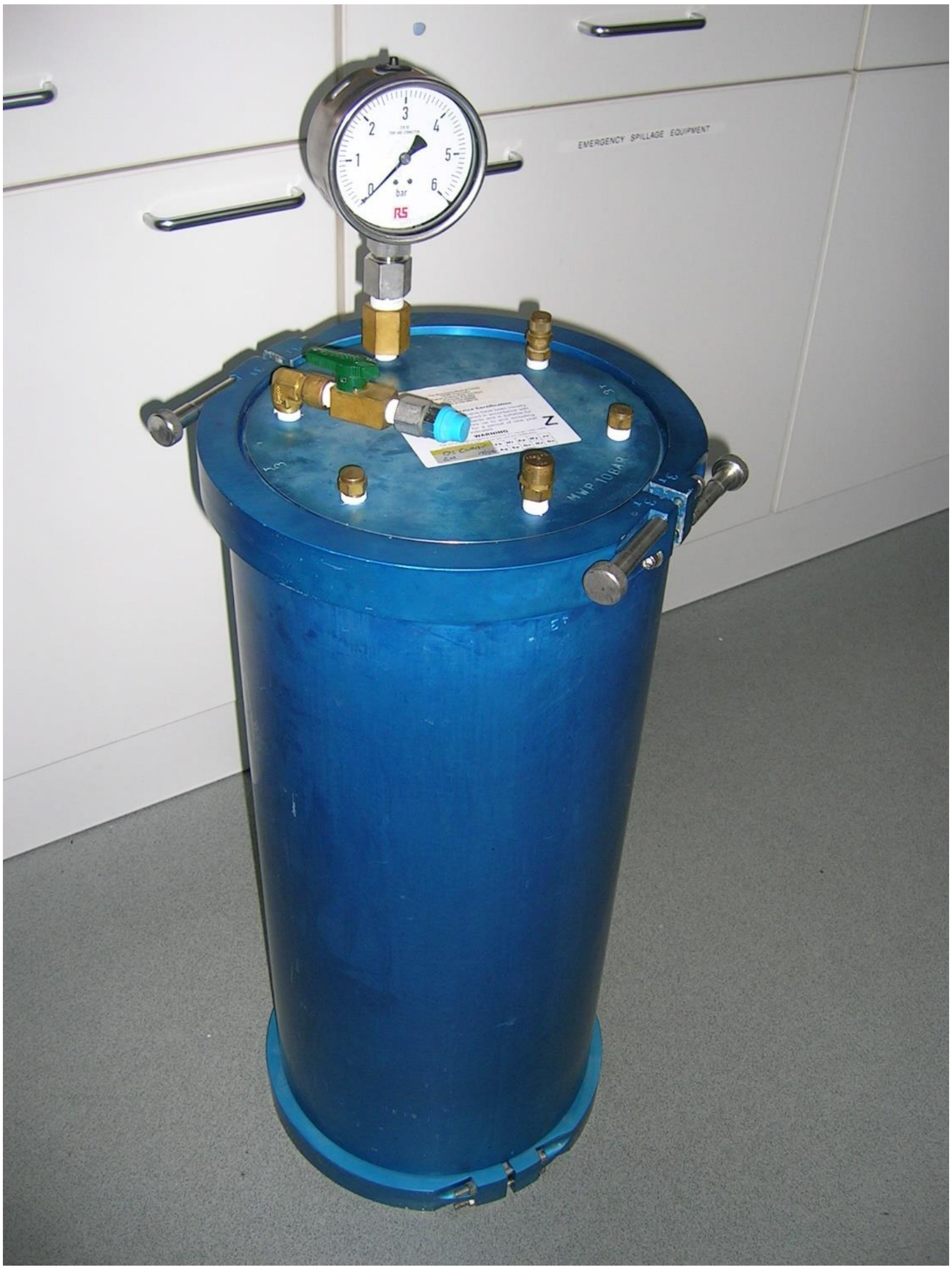

Figure S5 – Chamber used to culture bacteria under increased atmospheric pressure

Table 3 – Samples collection metadata for sponges used in this study

Cruise	ROV	ROV Dive	Sample ID	Date	Latitude	Longitude	Depth (m)	Pressure (bar)	Temp°C
JC136	Isis	295	JC136_135	6/16/2016	58.8546378	-13.3993737	1051	107	6.39
JC136	Isis	295	JC136_134	6/16/2016	58.8546885	-13.3994155	1051	107	6.39
JC136	Isis	295	JC136_125	6/16/2016	58.8547103	-13.3994087	1051	107	6.39
GRNL2017	Holland I	495	GRNL_081	7/20/2017	54.1867235	-12.8472535	2228	225	3.49
GRNL2017	Holland I	495	GRNL_082	7/20/2017	54.18651317	-12.847798	2175	220	3.5
CE19015	Holland I	683	Sponge_009	8/13/2019	49.534534	-12.10624333	1208	122	6
CE19015	Holland I	683	Sponge_010	8/13/2019	49.53452567	-12.10614217	1208	122	6

Table 4 – Genbank accession numbers for strains reported in this study

#Accession	Sequence ID	Release Date
MZ723441	Bacillus_pumilus_strain_EE112_P4	"Aug 13, 2021"
MZ723442	Methylobacterium_goesingense_strain_PAMC_29342	"Aug 13, 2021"
MZ723443	Pseudomonas_sp_JHX-241_1	"Aug 13, 2021"
MZ723444	Pseudomonas_sp_JHX-241_2	"Aug 13, 2021"
MZ723445	Brevibacterium_frigoritolerans_strain_ER52	"Aug 13, 2021"
MZ723446	Psychrobacter_piscatorii_strain_EnD-2	"Aug 13, 2021"
MZ723447	Psychrobacter_sp_4-Z18	"Aug 13, 2021"
MZ723448	Uncultured_bacterium_clone_7A_11-051	"Aug 13, 2021"
MZ723449	Rhodococcus_sp_SS51_7	"Aug 13, 2021"
MZ723450	Psychrobacter_sp_JXH-75_1	"Aug 13, 2021"
MZ723451	Rhodococcus_yunnanensis_strain_YIM_70056	"Aug 13, 2021"
MZ723452	Micromonospora_tulbaghiae_strain_Pw20-195	"Aug 13, 2021"
MZ723453	Micrococcus_antarcticus_strain_XH180	"Aug 13, 2021"
MZ723454	Bacillus_algicola_strain_HMF_4132	"Aug 13, 2021"
MZ723455	Psychrobacter_sp_strain_AHE_PA_1	"Aug 13, 2021"
MZ723456	Psychrobacter_sp_JXH-75_2	"Aug 13, 2021"
MZ723457	Pseudomonas_sp_JHX-241_3	"Aug 13, 2021"
MZ723458	Psychrobacter_sp_strain_CJKOP-63_1	"Aug 13, 2021"
MZ723459	Kocuria_indica_strain_MS51	"Aug 13, 2021"
MZ723460	Erythrobacter_sp_strain_LA324	"Aug 13, 2021"
MZ723461	Psychrobacter_sp_strain_CJKOP-63_2	"Aug 13, 2021"
MZ723462	Dermacoccus_nishinomiyaensis_strain_BCX_20	"Aug 13, 2021"
MZ723463	Bacillus_sp_ITP29	"Aug 13, 2021"
MZ723464	Psychrobacter_sp_strain_AHE_PA_2	"Aug 13, 2021"
MZ723465	Pseudomonas_sp_JHX-241_4	"Aug 13, 2021"
MZ723466	Brevibacterium_frigoritolerans_strain_F124	"Aug 13, 2021"
MZ723467	Uncultured_bacterium_clone_Md-9	"Aug 13, 2021"
MZ723468	Pseudomonas_sp_strain_IMBM09_1	"Aug 13, 2021"
MZ723469	Erythrobacter_sp_strain_A6_0	"Aug 13, 2021"
MZ723470	Dietzia_sp_strain_H0B	"Aug 13, 2021"
MZ723471	Psychrobacter_sp_strain_CKJOP-63	"Aug 13, 2021"
MZ723472	Pseudomonas_stutzeri_strain_2Dphe2	"Aug 13, 2021"
MZ723473	Psychrobacter_sp_strain_AHE_PA_3	"Aug 13, 2021"

MZ723474	Microbacterium_maritypicum_strain_P-BL63	"Aug 13, 2021"
MZ723475	Bacillus_sp_strain_MJS-AB-C4	"Aug 13, 2021"
MZ723476	Bacillus_toyonensis_strain_WS1-2	"Aug 13, 2021"
MZ723477	Dietzia_psychralcaliphila_strain_Y96	"Aug 13, 2021"
MZ723478	Rhodococcus_sp_HLSB305_2	"Aug 13, 2021"
MZ723479	Pseudomonas_sp_strain_IMBM09_2	"Aug 13, 2021"

References

Bowman, J. P. (2001) 'Methods for psychrophilic bacteria', *Methods in Microbiology*, pp. 591–614.

Cho, J. C. and Giovannoni, S. J. (2004) 'Cultivation and Growth Characteristics of a Diverse Group of Oligotrophic Marine Gammaproteobacteria', *Applied and Environmental Microbiology*, 70(1), pp. 432-440.2004.

Kato, S. *et al.* (2018) 'Isolation of previously uncultured slowgrowing bacteria by using a simple modification in the preparation of agar media', *Applied and Environmental Microbiology*, 84(19).

Keren, R., Lavy, A. and Ilan, M. (2016) 'Increasing the Richness of Culturable Arsenic-Tolerant Bacteria from Theonella swinhoei by Addition of Sponge Skeleton to the Growth Medium', Microbial Ecology, 71(4), pp. 873–886.

Margassery, L. M. *et al.* (2012) 'Diversity and antibacterial activity of bacteria isolated from the coastal marine sponges Amphilectus fucorum and Eurypon major', *Letters in Applied Microbiology*, 55(1), pp. 2–8.

Sipkema, D. *et al.* (2011) 'Multiple approaches to enhance the cultivability of bacteria associated with the marine sponge Haliclona (gellius) sp', *Applied and Environmental Microbiology*, 77(6), pp. 2130–2140.
